# Raised Intraocular Pressure as a Potential Risk Factor for Visual Loss in Leber Hereditary Optic Neuropathy

**DOI:** 10.1371/journal.pone.0063446

**Published:** 2013-05-07

**Authors:** Anais Thouin, Philip G. Griffiths, Gavin Hudson, Patrick F. Chinnery, Patrick Yu-Wai-Man

**Affiliations:** 1 Department of Neurology, Royal Victoria Infirmary, Newcastle upon Tyne, United Kingdom; 2 Institute of Neuroscience, Newcastle University, Newcastle upon Tyne, United Kingdom; 3 Department of Ophthalmology, Royal Victoria Infirmary, Newcastle upon Tyne, United Kingdom; 4 Wellcome Trust Centre for Mitochondrial Research, Institute of Genetic Medicine, Newcastle University, Newcastle upon Tyne, United Kingdom; University of Perugia, Italy

## Abstract

Leber Hereditary Optic Neuropathy (LHON) is an important cause of inherited mitochondrial blindness among young adults. The majority of patients carry one of three mitochondrial DNA (mtDNA) point mutations: m.3460G>A, m.11778G>A and m.14484T>C, all of which affect critical complex I subunits of the mitochondrial respiratory chain. LHON is characterised by marked incomplete penetrance, clearly implying that the mtDNA mutation is insufficient on its own to trigger retinal ganglion cell dysfunction and visual loss. In this case series of three affected patients harbouring the m.11778G>A mutation, we provide evidence suggesting that raised intraocular pressure could be a risk factor triggering visual loss in at-risk LHON carriers.

## Introduction

Leber Hereditary Optic Neuropathy (LHON) is a primary mitochondrial DNA (mtDNA) disorder that classically presents in the second and third decades of life with bilateral, subacute, severe visual loss [1], [2]. The visual prognosis is poor and the majority of patients remain significantly visually impaired. About 90% of patients will harbour one of three common causative mtDNA mutations: m.3460G>A, m.11778G>A, and m.14484T>C [3]. An intriguing, and as yet unexplained, aspect of LHON is the marked incomplete penetrance observed in this mitochondrial disorder, with only ∼50% of male carriers and ∼10% of female carriers experiencing visual loss during their lifetime [1], [2]. The mtDNA mutation is clearly insufficient on its own and a number of secondary nuclear genetic factors and environmental triggers have been implicated to account for this disparity. In a recent large study of 125 independent LHON families, smoking was strongly associated with an increased risk of visual loss and a convincing biological trend was observed when comparing light and heavy smokers [4]. There are also a number of published cases reports where disease conversion seemed to be temporally related to insults such as head trauma [5]–[7], occupational inhalation of chemical toxins [8], or secondary to iatrogenic exposure to antituberculous or antiretroviral drugs [9]–[11]. Although these causal links are difficult to prove unequivocally, the identification of modifiable environmental triggers is especially important given the current paucity of effective treatment strategies in LHON [1], [2]. In this case series of three affected patients carrying the m.11778G>A mtDNA mutation, we provide evidence suggesting that raised intraocular pressure could be an exacerbating risk factor precipitating retinal ganglion cell (RGC) dysfunction and the onset of visual loss in susceptible LHON carriers.

## Report of Cases

### Case 1

**Figure 1:**
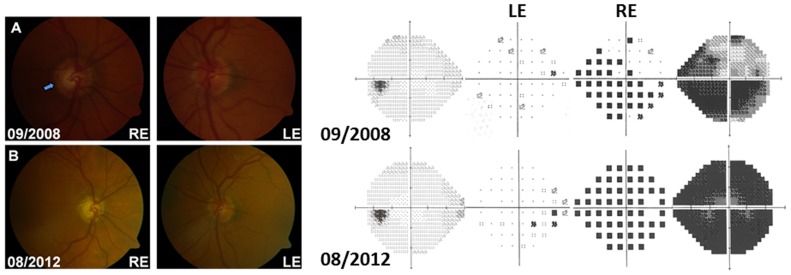
Optic disc appearance and visual fields of Patient 1. (A) Right optic disc cupping (arrow) was noted when the patient first presented with visual loss in the right eye. The left optic disc was normal. Humphrey™ visual field perimetry was carried out using the 24-2 SITA-FAST protocol. The right visual field showed a caecocentral scotoma with superior and inferior nasal step defects, in keeping with glaucomatous optic nerve damage prior to LHON disease onset (Mean deviation in September 2008: LE = −2.48 dB, RE = −22.06 dB). (B) Progression of right optic disc cupping with pallor of the remaining neuroretinal rim. The left optic disc remained normal. A dense field defect was present in the right eye (Mean deviation in August 2012: LE = −2.78 dB, RE = −29.25 dB). The changes in peripapillary retinal nerve fibre layer thickness over that period of time have been provided in ***Figure S1***.

A 63-year-old white man presented with sudden-onset, painless visual loss in his right eye down to 6/60 (**Table 1**). Except for a longstanding, divergent, amblyopic left eye (6/18), there was no other significant past medical history. There was no family history of early-onset visual loss. He was a long-term smoker with a daily consumption of 10–15 cigars. At initial presentation, the intraocular pressure was found to be markedly elevated in the right eye (43 mmHg), but within the normal range in the left eye (14 mmHg). The cornea was clear and gonioscopy showed wide-open drainage angles. There was a right relative afferent pupillary defect and a marked reduction in colour vision in the right eye. Asymmetric cupping of the right optic disc was noted on fundus examination (**Figure 1**). Fluorescein angiography was normal with no evidence of optic disc leakage or vasculitis. The patient was started on ocular anti-hypertensive treatment, but despite adequate intraocular pressure control being achieved, visual acuity in the right eye deteriorated further to count fingers over a one-month period. The patient was subsequently referred to the neuro-ophthalmology service and he was extensively investigated. Magnetic resonance imaging (MRI) of the brain and orbit was normal with no enhancement of the anterior visual pathways noted with gadolinium contrast. Rather surprisingly, molecular genetic testing eventually revealed the presence of a homoplasmic m.11778G>A LHON mutation as the cause of this patient’s atypical, unilateral optic neuropathy. The patient’s left eye has remained uninvolved over a four-year follow-up period.

**Table 1:** Clinical features and management of reported LHON cases.

Case	Trauma/Drugs^a^	Eye	Pre-visual loss			LHON disease onset			Last follow-up visit		
			IOP	VA	Treatment	IOP	VA	Treatment	IOP	VA	Treatment
**1**	No	RE	N/A	6/6		43	6/60	g. latanoprost nocte (RE) was started initially, but then discontinued because of suboptimal treatment effect	16	6/36	g. latanoprost nocte (RE) was re-introduced in the interim as IOPs over 16 mmHg were frequently recorded during the patient’s early follow-up visits
		LE^b^	N/A	6/18		14	6/18	Fixed combination g. dorzolamide 2% with g. timolol 0.5% bd (RE) was introduced with a satisfactory drop in IOP achieved	15	6/12	IOP measurements in the LE remained consistently below 16 mmHg without treatment
								Recruited into the RHODOS LHON trial and randomized to the active treatment arm (Idebenone 300 mg tds for 24 weeks) in 2009			Co-enzyme Q10 120 mg bd was prescribed by the patient’s family physician at his request (April 2011– September 2012)
**2**	No	RE	34	6/9	Raised IOPs were first documented 16 months before the onset of visual loss	16	6/60	Visual deterioration in the LE started 2 months after disease conversion in the RE	16	CF	g. latanoprost nocte (OU), g dorzolamide 2% bd (OU), and g. timolol 0.5% bd OU
		LE	40	6/6	g. latanoprost nocte (OU) was started for treatment of OHT with a reduction in IOPs in the mid-20s range (24–26 mmHg)	16	6/9	g. latanoprost nocte (OU) and g. dorzolamide 2% tds (OU)	16	CF	g. timolol 0.5% bd OU was introduced 11 months earlier by the patient’s glaucoma specialist
					Evidence of glaucomatous progression was noted in the LE 2 months before the onset of visual loss (IOPs 26 mmHg OU), with inferonasal notching of the neuroretinal rim (**Figure 2A**)						
					g. dorzolamide 2% tds (OU) was then introduced to achieve a lower target IOP						
**3**	No	RE	30	6/12	Raised IOPs were first documented 12 months before the onset of visual loss	18	3/60	Visual deterioration in the LE started 9 months after disease onset in the RE	12	6/9	g. bimatoprost nocte (OU) and fixed combination g. dorzolamide 2% with g. timolol 0.5% bd (OU)
		LE	30	6/6	g. bimatoprost nocte (OU) was started for treatment of glaucoma with a reduction in IOPs in the high teens range	18	6/6	Fixed combination g. dorzolamide 2% with g. timolol 0.5% bd (RE) was introduced to achieve lower target IOPs	12	6/60	Argon laser trabeculoplasty (OU) was performed in October 2010 by the patient’s glaucoma specialist
								Idebenone 300 mg tds was started shortly after molecular confirmation of the m.11778G>A LHON mutation			Idebenone 300 mg tds – no adverse reactions have been experienced so far

a Antituberculous and antiretroviral treatment;

b Amblyopic eye with a divergent strabismus; CF: count fingers; IOP: intraocular pressure; LE: left eye; mg: milligrams; N/A: not available; OHT: ocular hypertension; OU: both eyes; RE: right eye; tds: three times a day; VA: visual acuity.

### Case 2

**Figure 2:**
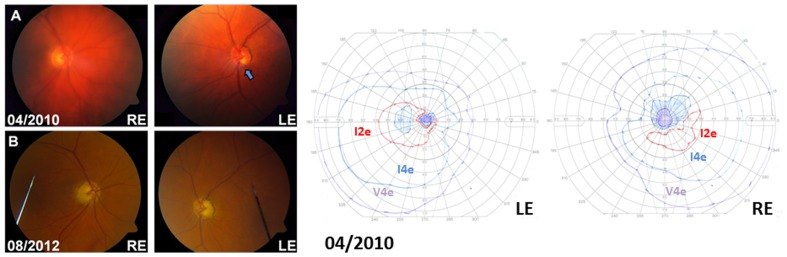
Optic disc appearance and visual fields of Patient 2. (A) Inferotemporal notching of the left optic disc was present at LHON disease onset (arrow). The patient was unable to perform Humphrey™ visual field perimetry reliably. Goldmann visual fields showed a steep-sided caecocentral scotoma in the right eye and a central scotoma in the left eye. (B) Advanced bilateral optic disc cupping with pallor of the remaining neuroretinal rim. Although visual field assessment became increasingly difficult as the patient’s visual acuities deteriorated to count fingers in both eyes, gradual peripheral field constriction was noted during the course of his follow-up visits. The changes in peripapillary retinal nerve fibre layer thickness over that period of time have been provided in ***Figure S2***.

A 72-year-old white man had been under regular ophthalmological review for bilateral ocular hypertension (**Table 1**). He was a non-smoker and there was no significant past medical or family history. Satisfactory intraocular pressure control proved difficult to achieve and glaucomatous progression in the left eye, with intraocular pressures of 26 mmHg in both eyes, was documented two months prior to LHON disease onset (**Figure 2**). At the time of disease conversion, the patient became aware of painless visual loss in his right eye over a two-week period. His best-corrected visual acuities were 6/60 in the right eye and 6/9 in the left eye when he first presented to the emergency department. Visual deterioration in the left eye started two months later and at the visual nadir, the patient could only perceive hand movements bilaterally. Despite normal inflammatory markers and a low index of suspicion for giant cell arteritis, the patient was started on oral prednisolone (60 milligrams, once a day). No treatment effect was observed and the steroid dose was rapidly tapered off. The patient was admitted under the neuro-ophthalmology team for further investigation. Neuroimaging was unremarkable and a lumbar puncture showed normal cerebrospinal fluid constituents. LHON was considered in the differential diagnosis and this was confirmed by the identification of a homoplasmic m.11778G>A LHON mutation.

### Case 3

**Figure 3:**
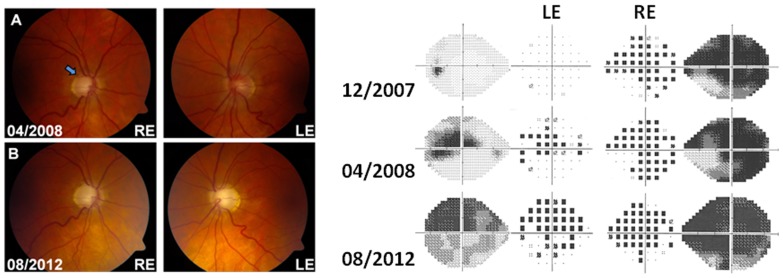
Optic disc appearance and visual fields of Patient 3. (A) Advanced cupping of the right disc with a small peripapillary haemorrhage still persisting at the superotemporal disc margin (arrow). The patient had been aware of visual deterioration in the right eye since April 2007. Visual loss started in the left eye in January 2008. (B) Advanced bilateral optic disc cupping with pallor of the remaining neuroretinal rim. The right panel shows the progression of the visual field defects in both eyes at three different time points: (i) after the onset of visual loss in the right eye (Mean deviation in December 2007: LE = −1.05 dB, RE = −25.10 dB); (ii) following disease onset in the fellow eye (Mean deviation in April 2008: LE = −9.03 dB, RE = −22.97 dB); and (iii) at the patient’s last follow-up visit (Mean deviation in August 2012: LE = −17.03 dB, RE = −27.40 dB). The changes in peripapillary retinal nerve fibre layer thickness over that period of time have been provided in ***Figure S3***.

A 58-year-old white man was being managed by the glaucoma team for right-sided advanced glaucoma and left-sided ocular hypertension (**Table 1**). The right optic disc was already deeply excavated when the diagnosis of primary open angle glaucoma was first made. He was a non-smoker and there was no significant past medical or family history. One year after raised intraocular pressures were first recorded, the patient reported painless visual loss in his right eye over a one-month period down to 3/60 (**Figure 3**). A peripapillary haemorrhage was present at the superotemporal disc margin and the rapid visual deterioration was ascribed to end-stage glaucomatous damage in an already severely compromised optic nerve. Nine months later, the patient experienced rapid visual loss in his left eye with an expanding dense central scotoma. At the nadir, the patient’s best-corrected visual acuities were 3/60 bilaterally. MRI of the brain and orbit with gadolinium contrast enhancement was normal. A neuro-ophthalmological second opinion was sought and the patient was found to harbour a homoplasmic m.11778G>A LHON mutation during his investigative work-up. He self-initiated treatment with idebenone (300 milligrams, three times a day) shortly after the molecular diagnosis was made (**Table 1**). The visual acuity in his right eye gradually improved to 6/9 over the following two-year period and the patient has opted to remain on long-term idebenone treatment. This study had the relevant institutional ethical approval (County Durham & Tees Valley 1 Research Ethics Committee, 08/H0905/106) and it was carried out in compliance with the Declaration of Helsinki. Participants provided their written consent for genetic testing and participation into this study.

## Discussion

Irrespective of sex and mutational status, the majority of LHON carriers lose vision before the age of 50 years and this epidemiological observation has been confirmed in different geographical populations [1], [2]. The three patients in this report therefore represent relatively late-onset cases, raising some rather intriguing questions as to the possible triggers for visual loss in these otherwise healthy m.11778G>A mutation carriers. Unilateral optic neuropathy has been described only rarely in LHON [6], [12]–[15], and it is tempting to draw a possible causal link between the atypical, unilateral clinical course in Patient 1 and the finding of a markedly raised intraocular pressure in the affected right eye. Over a four-year follow-up period, the left eye has remained uninvolved with normal intraocular pressures without treatment. Patients 2 and 3 were being actively managed for bilateral raised intraocular pressures before the onset of visual loss from LHON. For Patient 2, intraocular pressure control had been suboptimal and glaucomatous cupping of the left optic disc was documented two months before LHON disease conversion. Patient 3 had advanced optic disc cupping and significant field loss in his right eye when he was first diagnosed with primary open angle glaucoma. The rapid visual deterioration that developed a year later in the same eye was thought to be due to end-stage central RGC loss, before involvement of the fellow eye raised the suspicion of an alternative disease process. In all three LHON patients, disease conversion therefore occurred on the background of raised intraocular pressure, ongoing glaucomatous optic nerve damage, or both. LHON is a complex multifactorial disease with the primary causative mtDNA mutations being insufficient on their own to precipitate visual loss. We therefore cannot exclude the possibility that other secondary genetic and environmental risk modifiers could have played contributory roles in the atypical presentations of our three LHON cases, in addition to raised intraocular pressure.

LHON patients invariably remain with the legal requirement for blind registration as significant visual recovery is rare in this mitochondrial disorder [16]. If it does occur, the improvement in visual function is frequently modest with clearing islands of vision developing slowly within the central scotoma (fenestrations) typically in the first year after disease onset [1], [2]. This pattern of visual recovery is much more likely among young carriers affected before the age of 20 years and with the m.14484T>C mutation, which carries the best prognosis compared with the other two primary LHON mutations [1], [2]. The significant improvement in central visual acuity observed in the right eye of Patient 3 is therefore unusual, especially in the context of the m.11778G>A mutation. Although no visual recovery occurred in the left eye, the natural history could have been positively influenced by the initiation of idebenone treatment, which has shown promise as a possible neuroprotective agent in a recently completed randomised placebo-controlled trial [17].

*How could raised intraocular pressure be linked with an increased risk of visual loss in LHON?* RGC loss in this mitochondrial optic neuropathy is ultimately the consequence of an underlying, self-perpetuating bioenergetic deficit [18]. Raised intraocular pressure could exacerbate the precarious homeostatic state prevailing in RGCs carrying a pathogenic LHON mutation by further impeding axoplasmic flow or by impairing the vascular supply at the optic nerve head and the critical transition region spanning the lamina cribosa. The parvocellular RGCs within the papillomacular bundle have relatively small cross-sectional areas compared with the larger magnocellular RGC population [19], [20]. This anatomical factor is likely to impose additional physical constraints to axoplasmic flow under pathological conditions, such as raised intraocular pressure, particularly in the highly energy-dependent, unmyelinated segment of the optic nerve. There is also mounting evidence that mitochondrial dysfunction is a key element in the pathophysiology of glaucomatous optic neuropathy [21]–[23]. Raised intraocular pressure has been shown to induce fragmentation of the mitochondrial network within RGC axons, which further compromises the efficiency of the mitochondrial respiratory chain and leads to the release of pro-apoptotic cytochrome *c* molecules [24], [25].

*Could the postulated link between raised intraocular pressure and visual loss in LHON be a chance association?* We have estimated the odds of a male LHON carrier losing vision beyond the age of 50 years and also having elevated intraocular pressures over 30 mmHg at 1 in 33.8 million (**Table 2**). The affected LHON patients in this case series all originate from the North of England, which encompasses a catchment population of about three million individuals. The identification of three such atypical cases in this defined geographical region is therefore more than what would be expected by chance alone. Interestingly, *Nucci and colleagues* have recently described a 53-year-old woman with pre-existing primary open angle glaucoma who developed bilateral sequential visual loss five years after she was first diagnosed [26]. Similar to our own case series, the patient had a number of unusual features that initially confounded the underlying reason for her dramatic visual deterioration; with a relatively late age of onset, no obvious environmental triggers, and the absence of a maternal family history of blindness. In addition, LHON is marked by a marked sex bias with female carriers having a significantly lower risk of disease conversion compared with male carriers. Although the case report by *Nucci and colleagues* and our point estimates (**Table 2**) both support a plausible pathological link, our hypothesis that raised intraocular pressure could precipitate disease conversion in LHON needs to be substantiated further and the recent availability of *in vivo* disease models provides tractable experimental means of verifying this [27], [28].

**Table 2:** Chance occurrence of raised intraocular pressure ≥30 mmHg in an affected LHON carrier.

Variable		Odds	Reference
a	Carrier of one of three primary mtDNA LHON mutations (m.3460G>A, m.11778G>A, and m.14484T>C)	1 in 8,460	[32]
b	Lifetime risk of visual loss for a male LHON carrier	1 in 2	[1], [2]
c	Disease conversion in a LHON carrier >50 years old	1 in 20	[32]
d	Intraocular pressure ≥30 mmHg in an individual >50 years old	1 in 100	[33]
	Chance co-occurrence of above variables (a×b×c×d)	1 in 33.8M	

Various treatment algorithms have been proposed for the management of ocular hypertension and primary open angle glaucoma, including the target intraocular pressure that needs be achieved [29]–[31]. Until further evidence becomes available, it seems reasonable to set a lower threshold for initiating treatment for LHON carriers given the possible deleterious consequences of raised intraocular pressure on mitochondrial function and RGC survival.

## Supporting Information

Figure S1 **Changes in peripapillary retinal nerve fibre layer thickness for Patient 1.** (A) April 2009: average thickness OD = 47.15 µm, OS = 91.12 µm; (B) August 2012: average thickness OD = 59 µm, OS = 89 µm. Measurements were carried out either with the Fast RNFL (3.4) acquisition protocol on a time-domain Stratus OCT™ (Carl Zeiss Meditec, Dublin, CA), or the high-resolution spectral-domain Cirrus™ (Carl Zeiss Meditec, Dublin, CA).
(TIF)

Figure S2 **Changes in peripapillary retinal nerve fibre layer thickness for Patient 2.** (A) April 2010: average thickness OD = 84.86 µm, OS = 76.53 µm; (B) August 2012: average thickness OD = 57 µm, OS = 69 µm.
(TIF)

Figure S3 **Changes in peripapillary retinal nerve fibre layer thickness for Patient 3.** (A) April 2008: average thickness OD = 61.47 µm, OS = 97.42 µm; (B) August 2012: average thickness OD = 66 µm, OS = 73 µm.
(TIF)
