# Supplementary figures and images for: Raised Intraocular Pressure as a Potential Risk Factor for Visual Loss in Leber Hereditary Optic Neuropathy
Source: PLoS One. 2013 May 7;8(5):e63446. doi: 10.1371/journal.pone.0063446 (PMC3646743; doi:10.1371/journal.pone.0063446)

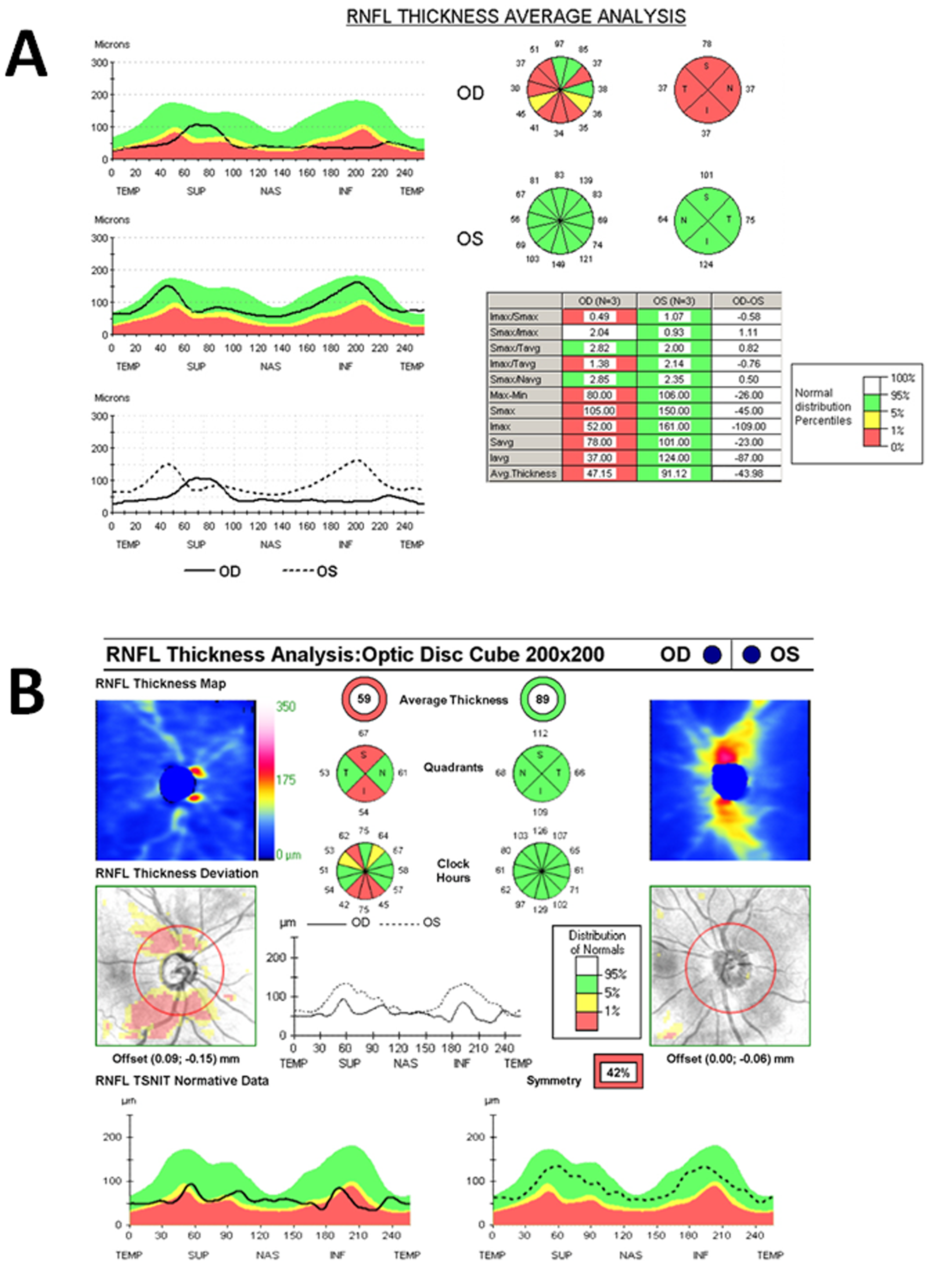

Supplement: Figure S1 — Changes in peripapillary retinal nerve fibre layer thickness for Patient 1. (A) April 2009: average thickness OD = 47.15 µm, OS = 91.12 µm; (B) August 2012: average thickness OD = 59 µm, OS = 89 µm. Measurements were carried out either with the Fast RNFL (3.4) acquisition protocol on a time-domain Stratus OCT™ (Carl Zeiss Meditec, Dublin, CA), or the high-resolution spectral-domain Cirrus™ (Carl Zeiss Meditec, Dublin, CA). (TIF) [file pone.0063446.s001.tif]

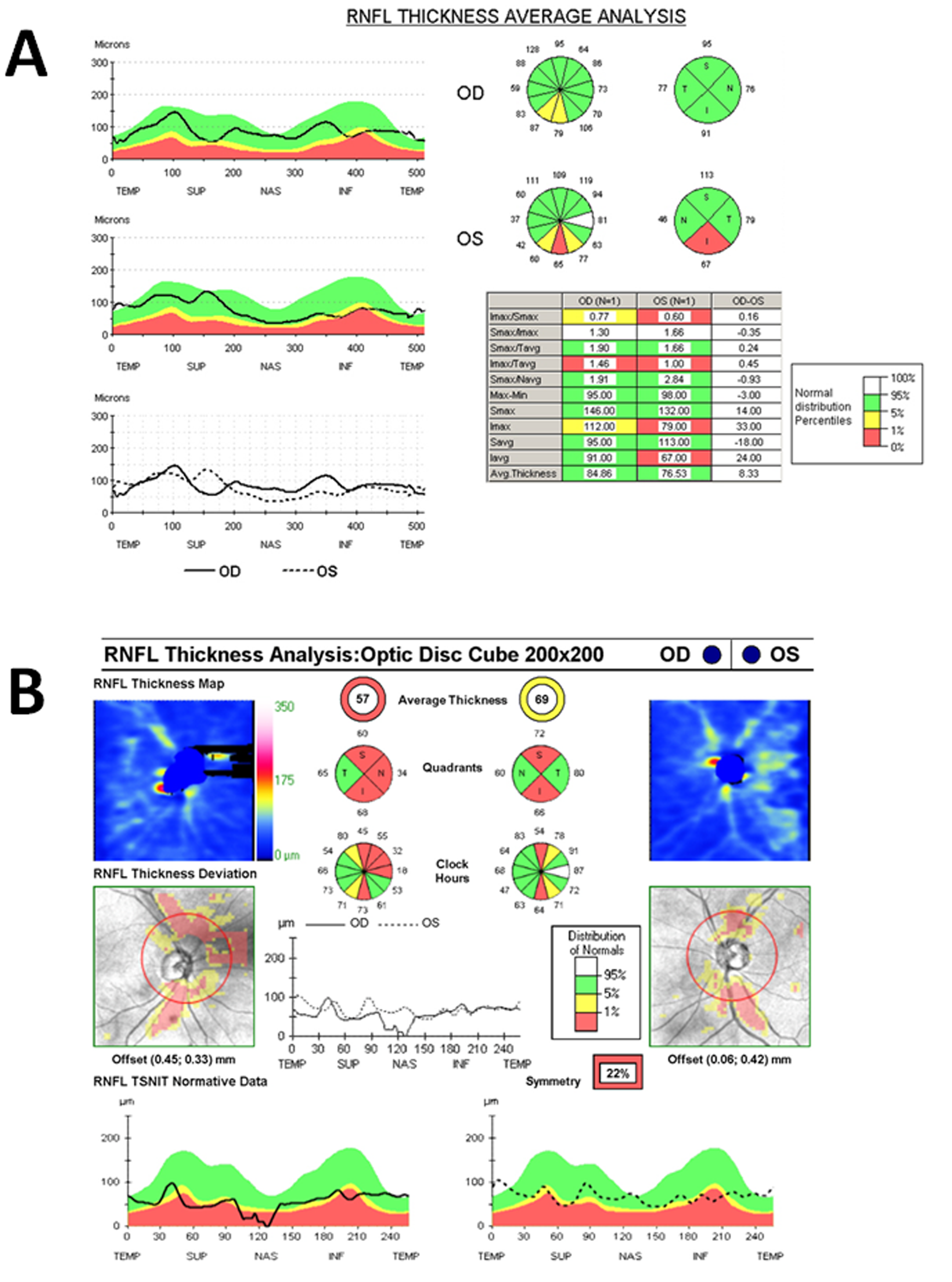

Supplement: Figure S2 — Changes in peripapillary retinal nerve fibre layer thickness for Patient 2. (A) April 2010: average thickness OD = 84.86 µm, OS = 76.53 µm; (B) August 2012: average thickness OD = 57 µm, OS = 69 µm. (TIF) [file pone.0063446.s002.tif]

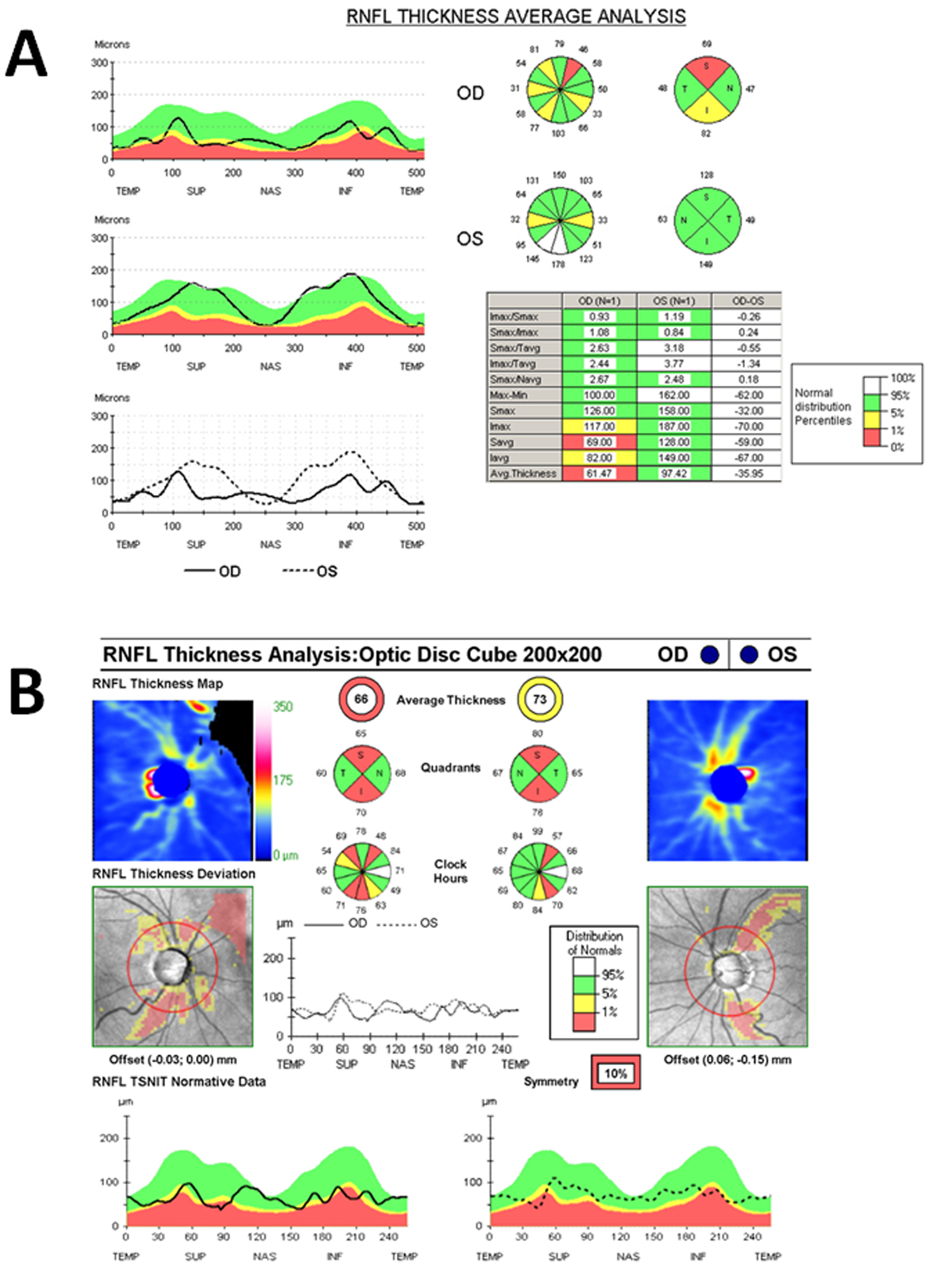

Supplement: Figure S3 — Changes in peripapillary retinal nerve fibre layer thickness for Patient 3. (A) April 2008: average thickness OD = 61.47 µm, OS = 97.42 µm; (B) August 2012: average thickness OD = 66 µm, OS = 73 µm. (TIF) [file pone.0063446.s003.tif]
